# Neurotoxicity-sparing radiotherapy for brain metastases in breast cancer: a narrative review

**DOI:** 10.3389/fonc.2023.1215426

**Published:** 2024-02-02

**Authors:** Dagmara Buczek, Renata Zaucha, Jacek Jassem

**Affiliations:** Department of Oncology and Radiotherapy, Medical University of Gdańsk, Gdańsk, Poland

**Keywords:** brain metastases, radiation-related neurotoxicity, hippocampus-avoiding radiotherapy, radiosurgery, cognitive function

## Abstract

Breast cancer brain metastasis (BCBM) has a devastating impact on patient survival, cognitive function and quality of life. Radiotherapy remains the standard management of BM but may result in considerable neurotoxicity. Herein, we describe the current knowledge on methods for reducing radiation-induced cognitive dysfunction in patients with BCBM. A better understanding of the biology and molecular underpinnings of BCBM, as well as more sophisticated prognostic models and individualized treatment approaches, have appeared to enable more effective neuroprotection. The therapeutic armamentarium has expanded from surgery and whole-brain radiotherapy to stereotactic radiosurgery, targeted therapies and immunotherapies, used sequentially or in combination. Advances in neuroimaging have allowed more accurate screening for intracranial metastases, precise targeting of intracranial lesions and the differentiation of the effects of treatment from disease progression. The availability of numerous treatment options for patients with BCBM and multidisciplinary approaches have led to personalized treatment and improved therapeutic outcomes. Ongoing studies may define the optimal sequencing of available and emerging treatment options for patients with BCBM.

## Simple summary

Up to 10-30% of breast cancer patients will develop metastases to the brain or meninges. Radiotherapy remains one of the main treatments for intracranial metastases. Although radiotherapy prolongs the survival of breast cancer patients, it is associated with considerable toxicity, which is particularly manifested in cognitive function deterioration. Animal studies suggest that these sequelae result mainly from radiation damage to the hippocampus. Avoiding this structure during brain radiotherapy and its pharmacological protection have been the subjects of multiple trials. Another brain-sparing approach that has emerged in the standard management of countable brain metastases is stereotactic radiotherapy. We present current knowledge on the efficacy and safety of these strategies in breast cancer patients with brain metastases.

## 1 Introduction

Breast cancer (BC) has surpassed lung cancer as the most commonly diagnosed malignancy among women (1). It is the second most common cause of malignant central nervous system (CNS) dissemination after lung cancer (2). According to the Surveillance, Epidemiology, and End Results Program, 10-30% of patients with BC develop brain or leptomeningeal metastases (BMs), making BMs the most common cause of BC-related deaths (3). New and more effective treatment methods, including anti-HER2 antibodies, CDK4/6 inhibitors, antibody−drug conjugates and immunotherapy, substantially prolong patients’ overall survival (OS), while the availability of precise CNS imaging increases the detection rate of asymptomatic BM (4). Currently, three options in BCBM treatment, i.e., systemic therapy, surgery and radiotherapy (RT), prolong OS by several months, mostly in patients with HER2-positive breast cancer (5). Owing to poor blood−brain barrier (BBB) penetration, chemotherapy has a limited role in the management of BM (6–8), but a better understanding of BM biology has facilitated the development of novel targeted therapies (9). In oligometastatic disease with fewer than five metastatic lesions, surgical excision and/or stereotactic radiosurgery (SRS) or stereotactic radiotherapy (SRT) are the treatments of choice (10–12). In patients with multiple or large BMs and uncontrolled extracranial disease, either whole-brain RT (WBRT) or partial-brain RT (PBRT) is used (13, 14). Although RT has significantly improved the survival of BCBM patients, a relatively large proportion of treated patients will develop radiation-related deterioration of cognitive functions (CFs), including deficits in memory, spatial information processing abilities, and learning difficulties that significantly affect their quality of life (15, 16). The incidence of radiation-induced brain damage is probably underestimated, but the use of novel magnetic resonance imaging (MRI) techniques, such as diffusion-weighted imaging, proton magnetic resonance spectroscopy, and perfusion MRI, has improved the ability to better characterize treatment-related changes. The frequency and severity of cognitive impairment following brain RT are affected by the patient’s age and education, tumor type, RT volume (WBRT, PBRT, SRS, and SRT), RT dose, time elapsed since treatment, definition of neurocognitive impairment, baseline neurocognitive function (NCF), and use of concurrent or previous chemotherapy, targeted agents or immunotherapy (17). We describe the current knowledge on the possibilities of neuroprotection in patients with BCBM receiving radiotherapy.

## 2 Local treatment for breast cancer brain metastases

For a long time, WBRT has been the standard of care for patients with BM since it improves the median OS from 1 to 6 months compared with best supportive care (7, 18, 19). Clinical studies have shown no difference in OS among various fractionation regimens. Radiosensitizers were also found to fail to improve the prognosis of patients with BM treated with RT (20–22). Therefore, WBRT of 30 Gy in 10 fractions or 20 Gy in 5 fractions has remained the standard of treatment, regardless of BM histology (13, 14, 23–25).

In the case of a single BM from multiple primaries, surgical excision plus WBRT was compared with WBRT alone in randomized trials (26–28), and the results were inconclusive. Combined therapy was shown to be better than WBRT alone (median survival 9-10 months versus 3-6 months) in two studies (26, 27), especially in patients without active systemic disease. Patients with BCBM constituted only 7.5-12% of the studies population and the only histological stratification was the division into patients with NSCLC vs. other sites of cancer and no statistical difference was found. Wroński et. al (29) reported retrospective series of 70 BCBM patients (10% of patients operated due to brain metastases between 1974 and 1993 in their hospitals) treated with neurosurgery with mOS=16.2 months from the time of diagnosis of BM and mOS=14 months from the time of surgery. Among the favorable prognostic factors, the authors mentioned younger age of patients, smaller size of metastases (but not their number) and hormonal status with mOS=21.9 months for ER+ vs. 12.5 months for ER- BC. However, in multivariate analysis, only the use of WBRT after surgery and the absence of meningeal involvement had good prognostic significance. In another retrospective series of 198 patients with BCBM, mOS=14.9 months was also found, but only in the group of 28% of patients with single BM treated surgically or with the use of a gamma knife (SRS), while patients undergoing WBRT had mOS=5.4 months (30). In a more recent series of 53 patients with BCBM from 1994-2010 treated with resection and in 2/3 cases with radiotherapy (SRS-gamma knife, WBRT or both), mOS was 16 months, even though 30% were patients with TNBC and 45% patients with HER2+, probably due to the greater use of effective systemic therapies (31).

On the one hand, the better prognosis of patients with a limited number of BMs has led to interest in more aggressive local treatment. In the RTOG 9508 randomized trial, in patients with 1-3 BMs, a stereotactic boost added to WBRT improved not only the survival of those with a single unresectable brain metastasis (mOS: 6.5 vs. 4.9 months, p=0.0393) but also the functional autonomy of all patients (stable or improved Karnofsky Performance Status [KPS] score at 6 months in 43% vs. 27% in the WBRT group, p=0.03) (32). On the other hand, in the European Organisation for Research and Treatment of Cancer EORTC 22952-26001 study, OS and functionally independent survival were similar, regardless of the addition of WBRT to SRS in the treatment of 1-3 BM (33). These results have changed the paradigm of adding localized treatment of BM to WBRT by using SRS without WBRT, with a single dose of radiation precisely delivered to the BM, maximizing the chance for local control, and sparing the normal brain tissue by omitting WBRT. Although BCBM patients constituted only 12% in the EORTC study, retrospective data confirm very good results of SRS in this group of patients with mOS=15.7 months (34). In the analysis of 91 patients with BCBM who received SRS, a statistically significant prognostic factors were: receptor status, BCBM volume and stable extracranial disease. Patients with ER+/HER2- had mOS=13.8 months, the best results were obtained in patients ER+/HER2+ with mOS=21.4 months and ER-/HER2+ with mOS=20.4 months, and the worst prognosis was reported in patients with TNBC with mOS=8.5 months. These results were better than overall survival reported in other retrospective series of patients with BCBM (35, 36). BCBM volume > 10 cm3 was associated with a worse prognosis with mOS=9.2 months. The authors suggested the possibility of selecting this group of patients for combined treatment with SRS + WBRT, although in the light of the latest knowledge, these patients may also be candidates for FSRT, but the value of both approaches requires validation. Patients with stable extracranial disease undergoing SRS for BCBM had a better prognosis (mOS=20.1 months) compared to patients with progressive extracerebral lesions (mOS=11.4 months). Interestingly, patients without extracranial disease had an mOS of 13.4 months, but this is probably due to the significant proportion of TNBC patients in this group, in whom BMs are often the first manifestation of dissemination and the prognosis remains unfavorable.

In the lack of randomized trials comparing the outcomes of SRS with or without WBRT in BCBM patients, we summarized phase III randomized trials including patients with different numbers (single, up to three, and up to four), sizes and pathological types of BM, as well as resected single BM (**Table 1**). Most of these studies have shown less cognitive impairment in patients with BM after SRS than after WBRT but at the cost of a higher risk of further intracranial progression. On the other hand, WBRT significantly decreased in-brain recurrences but increased the risk of death due to neurotoxicity in the setting of intact as well as resected BM (37, 38, 40, 41). Knowing that the mere presence of brain metastases causes cognitive disorders in patients with BM (42) and that the use of SRS/FSRT increases the risk of further brain metastases that aggravate these disorders and require further local treatment, the effects of which overlap with existing cognitive problems, it seems that the several months of observations in the cited studies (maximum 6 months) may be insufficient to assess long-term radiation-induced neurotoxicity.

**Table 1:** Phase III trials and meta-analyses of S/SRS+/-WBRT for the treatment of brain metastases (BMs) with cognitive endpoints.

Reference	Inclusion criteria	Number of patients	Primary endpoint	OS (months)	Outcome	Secondary endpoint
**Aoyama et al. (** 37) **(JROSG)**	**1-4 BMs** **(<3 cm each)**	**132**	**OS**	**SRS+WBRT: 7.5** **SRS alone: 8.0** **1-year actuarial survival rate:** **SRS+WBRT 38.5%** **SRS alone 28.4%** **(p=0.42)**	**No significant differences in systemic and neurological functional preservation and toxic effects of radiation** **(neurocognitive function assessed by the MMSE)**	**12-month brain tumor recurrence rate:** **SRS+WBRT 46.8%** **SRS alone 76.4%** **(p<0.001)** **Death of neurologic causes:** **SRS+WBRT 22.8%** **SRS alone 19.3%** **(p=0.64)**
**Chang et al. (** 38) **(MDACC)**	**1-3 BMs**	**58**	**5-point drop on the HVLT-R total recall at 4 months**	**SRS+WBRT: 5.7** **SRS alone: 15.2** **(p=0.003)**	**Neurocognitive decline at 4 months:** **SRS+WBRT 52%** **SRS alone 24%**	**Free from CNS recurrence at 1 year:** **SRS+WBRT 73%** **SRS alone 27%** **(p=0.0003)**
**Kocher et al. (** 33) **(EORTC 22952-26001 study)**	**1-3 BMs** **(surgery/SRS +/-WBRT)**	**359** **(199 SRS, 160 surgery)**	**WHO PS deterioration to more than 2**	**S/SRS+WBRT: 10.7** **S/SRS alone: 10.9** **(p=0.89)**	**The median time to WHO PS deterioration to more than 2:** **S/SRS+WBRT 9.5 months** **S/SRS alone 10 months** **(p=0.71)**	**2-year relapse rate at initial site:** **S alone 59%** **S+WBRT 27%** **(p<0.001)** **SRS alone 31%** **SRS+WBRT 19%** **(p=0.04)** **2-year relapse rate at new sites:** **S alone 42%** **S+WBRT 23%** **(p=0.008)** **SRS alone 48%** **SRS+WBRT 33%** **(p=0.023)** **Neurologic death rate:** **S/SRS+WBRT 28%** **S/SRS alone 44%** **(p<0.002)**
**Tsao et al. (** 11) **(meta-analysis)**	**1-4 BMs**	**190 pts for OS analysis** **389 pts for other analyses**	**OS, LC, DBC**	**OS:** **SRS alone vs. SRS+WBRT HR=0.98 (0.71-1.35)** **p=0.88**	**LC:** **SRS alone vs. SRS+WBRT HR=2.61 (1.68-4.06)** **p<0.0001** **DBC:** **SRS alone vs. SRS+WBRT HR=2.15 (1.55-2.99)** **p<0.00001**	**Neurocognition was not evaluated due to different tests used in included trials (the HVLT or MMSE)**
**Sahgal et al. (** 12) **(meta-analysis)**	**1-4 BMs**	**364**	**OS, LF, DBF**	**SRS+WBRT: 8.2 (4-13)** **SRS alone: 10.0 (4.5-18)**	**mLF:** **SRS+WBRT 7.4 months (3.8-16)** **SRS alone 6.6 months (3.4-14)** **mDBF:** **SRS+WBRT 6.5 months (3.8-16)** **SRS alone 4.7 months (2.8-11)**	**mOS <=50 years** **SRS+WBRT: 8.2 months** **SRS alone 13.6 months** **mOS > 50 years** **SRS+WBRT: 8.6 months** **SRS alone: 10.1 months** **DBF:** **<= 50 years: HR=0.9-1.43** **>50 years: HR=1.67-3.6** **Neurologic death – no significant differences:** **SRS+WBRT 25%** **SRS alone 30%**
**Brown et al. (** 39) **(Alliance study)**	**1-3 BMs**	**213**	**Decline > 1 SD from baseline on at least 1 cognitive test at 3 months (tests used: HVLRT-R immediate recall, HVLRT-R delayed recall, HVLRT-R recognition, Grooved Pegboard Test, COWAT, TMT-A, TMT-B)**	**SRS+WBRT: 7.4** **SRS alone: 10.4** **p=0.92**	**Cognitive deterioration:** **SRS+WBRT 91.7%** **SRS alone 63.5%** **(p<0.001)**	**QoL change from baseline** **SRS+WBRT: -12.0** **SRS alone: -1.0** **p=0.001** **Time to intracranial failure SRS vs. SRS+WBRT** **HR=3.6, p<0.001** **No significant difference in functional independence at 3 months**
**Kępka et al. (** 40) **(Polish study)**	**1 BM after resection**	**59**	**CINCF**	**2-year OS:** **S+WBRT 37%** **S+SRS 10%** **p=0.046**	**CINCF at 6 months was -8% in favor of WBRT (95% CI +17%- 35%; noninferiority margin: -20%)**	**Noninferiority of SRS after S was not demonstrated;** **2-year CIND rates in ITT analysis:** **S+WBRT 31%** **S+SRS 66%** **(p=0.015)**
**Brown et al. (** 41) **(NCCTG N107C/Cstudy)**	**1 resected BM with resected cavity < 5 cm** **(up to 3 unresected BMs were allowed)**	**194**	**OS, cognitive-deterioration- free survival** **(tests used: HVLRT-R immediate recall, HVLRT-R delayed recall, HVLRT-R recognition, Grooved Pegboard Test, COWAT, TMT-A, TMT-B)**	**S+WBRT: 11.6** **S+SRS: 12.2** **p=0.7**	**Cognitive-deterioration-free survival** **S+WBRT: 3.0 months** **S+SRS: 3.7 months** **p<0.0001**	**Cognitive deterioration at 6 months** **S+WBRT: 85%** **S+SRS: 52%** **p<0.00031**

JROSG, Japanese Radiation Oncology Study Group; OS, overall survival; MMSE, Mini-Mental State Examination; MDACC, M.D. Anderson Cancer Center; HVLT-R, Hopkins Verbal Learning Test-Revised; EORTC, European Organisation for Research and Treatment of Cancer; WHO, World Health Organization; PS, Performance Status; LC, local control; DBC, distant brain control; LF, local failure; DBF, distant brain failure; mLF, median local failure; mDBF, median distant brain failure; COWAT, Controlled Oral Word Association Test; TMT-A, Trail Making Test Part A; TMT-B, Trail Making Test Part B; QoL, quality of life; CINCF, Cumulative incidence of neurological/cognitive failure; CIND, Cumulative incidence of neurological death; NCCTG, North Central Cancer Treatment Group.

However, CF was prospectively assessed in only a minority of those trials using various tools and methods, such as the Hopkins Verbal Learning Test-Revised [HVLT-R] Immediate Recall, Delayed Recall and Recognition; Grooved Pegboard Test; Controlled Oral Word Association Test [COWAT]; Trail Making Test Part A [TMT-A]; and/or Trail Making Test Part B [TMT-B]. Owing to the heterogeneity of the patient populations, it is very difficult to compare these results and draw conclusions. The Mini-Mental State Examination (MMSE) showed very low sensitivity to detect RT-induced cognitive impairment (37). Using the Hopkins Verbal Learning Test-Revised (HVLT-R), Chang et al. (38) proved that combined SRS+WBRT vs. SRS alone in patients with 1-3 BMs improved the treatment outcomes at the cost of significant deterioration of learning and memory function as early as at 4 months.

Despite the doubts about SRS/FSRT, the lack of long-term survival benefit of adding WBRT established the role of SRS/FSRT in the treatment of oligometastatic brain metastases, especially in cancers such as breast cancer where metastasectomy/stereotactic radiotherapy of a limited number of metastases to other organs was already standard.

Promising results of SRS in oligometastatic BMs were extrapolated to polymetastatic BM treatment. The noninferiority of stereotactic radiotherapy in 5-10 vs. 2-4 BMs has been demonstrated in a nonrandomized study (HR=0.97, 95% CI, 0.81-1.18; p=0.78) (43, 44).

The development of molecular diagnostics and further research on the prognosis of patients with BM depending on prognostic factors led to the creation of the graded prognostic assessment (GPA) for estimating the survival of patients with brain metastases, including a separate breast GPA, the use of which shows the longer survival of patients with BCBM in relation to other patients with BM.

The prolonged survival of patients with BC due to advances in systemic treatment has led to an increased risk of late complications following WBRT, especially in those with the HER2-positive subtype, in whom intracranial spreading is particularly frequent (45, 46). Because of the lack of a survival advantage of WBRT added to stereotactic radiotherapy, SRS/FSRT alone is recommended for most patients, even in patients with multiple metastases, especially if their total volume is ≤15 ml (11, 43, 44, 47–49). The SRS dose and BM volume did not correlate with posttherapy CF (44, 46–49). Thanks to new technical solutions, SRS/FSRT for BM therapy has become more accessible and less time-consuming. Modern arc RT planning systems enable the creation of a single plan for several focal lesions in the brain (mono-isocenter technique), which, compared with the conventional multi-isocenter technique, not only shortens the treatment planning time but also decreases neurotoxicity by reducing the dose to the healthy brain (50).

## 3 Radiation-induced neurotoxicity

During standard radiation treatment (PBRT, WBRT, SRT, and SRS), healthy brain tissue is inevitably exposed to radiation. The exact mechanism underlying radiation-induced brain damage is not fully understood. For many years, the brain has been regarded as a highly radioresistant organ. Acute neurological symptoms were observed early after a single dose of 30 Gy, whereas white matter necrosis occurred after a conventionally fractionated dose of 60 Gy (51). With modern techniques, focal neurological deficits, epilepsy, and increased intracranial pressure have become less common (51, 52).

Early and early-delayed side effects, e.g., somnolence, headaches, drowsiness, attention deficits, and short-term memory loss, are caused by brain edema and transient demyelination, respectively. In contrast, late-delayed neurotoxicity is irreversible owing to white matter necrosis, vascular fibrosis, permanent demyelination, or gliosis (51). Biologically, these processes are associated with the proliferative capacity of glial and vascular endothelial cells. White matter necrosis is currently uncommon; however, the dynamic interplay among astrocytes, microglia, oligodendrocytes, endothelial cells, and neurons leads to radiation-induced neurocognitive dysfunction (16, 53). Most data on the mechanism of radiation-induced brain damage are obtained from studies on animal models, including rodents and nonhuman primates, and have suggested that the loss of hippocampal neurogenesis is a main problem (54, 55). Another hypothesis indicates that cognitive impairment after irradiation is caused by a neuroinflammatory cascade, including the disruption of the BBB, neural progenitor cell (NPC) death, hippocampal dysfunction, and direct activation of glia, causing the senescence-associated secretory phenotype (56).

### 3.1 Hippocampus

The hippocampus is the central player in memory and neurogenesis. Damage to this structure affects reasoning skills, learning difficulties, and memory and attention deficits and can progress to dementia. A preclinical study has shown that normal cognitive function (CF) is associated with neurogenesis in neural stem cells located in the subgranular zone of the hippocampal dentate gyrus (57). Transcriptomic analyses have shown that although hippocampal neurogenesis is decreased, it is preserved and possible in adulthood (57–59). Hippocampal glutamate receptor 1 and protein kinase C-gamma are likely responsible for synaptic plasticity in working memory (60). In mice, NPC maturation is suppressed or incorrect after ionizing radiation even at very low doses. Moreover, individuals exposed to irradiation did not pass the maze test, which is a hallmark of cognitive deterioration (52). Clinical observations have suggested similar effects in humans. Bilateral hippocampal irradiation with doses of >7.3 Gy was significantly correlated with cognitive dysfunction, with a 30% mean relative CF decrease at 4 months after RT (61).

### 3.2 Blood−brain barrier

For years, the BBB has been thought to be the most essential factor for BM resistance to systemic treatment. Abnormal tumor blood vessels facilitate the penetration of various molecules. As shown in an animal model, radiation destabilizes the plasma membrane of cells of the BBB and damages endothelial cells, increasing BBB permeability. The upregulation of proinflammatory genes and intracellular adhesion molecules leads to hypoxia, which amplifies the toxic changes in the irradiated brain microenvironment (62–64). Animal studies have shown that apoptosis observed 24 hours after WBRT leads to a 15% loss of cerebral microvascular endothelial cells (65).

### 3.3 Radiation-induced senescence

Radiation-induced DNA damage occurs directly from a high energy beam or indirectly via free radicals and reactive oxygen species. Single-strand DNA breaks may be repaired, while double-strand breaks are irreversible, causing cell apoptosis, senescence, mutations and genomic instability in astrocytes or neurons, endothelial cells, and fibroblasts. Thus, a complicated cascade of inflammatory processes in astrocytes involving cytokines, such as IL-6 and IL-1beta, changes the brain phenotype into a senescence-associated secretory phenotype (17, 66). The isoforms of p53 play a crucial role in promoting or restoring astrocyte senescence (17, 66).

### 3.4 Histological characteristics of radiation-induced brain injury

Every patient with neurological or neurocognitive changes requires evaluation to exclude the presence of a new BM, irradiated tumor progression, necrosis or other postradiation neurodegenerative changes. Despite progress in radiological imaging, no specific features allowing unequivocal differential diagnosis of degenerative versus cancer-related changes in the brain have been defined (67). Diagnosis in such cases may be achieved by neurosurgical excision, which is not possible in most patients. Tissue specimens from patients eligible for surgical excision usually contain residual tumor, necrotic tissue or both. Occasionally, viable normal brain tissue in a biopsy sample shows radiation-related changes, including astrogliosis, vascular alterations, tissue rarefaction, chronic inflammation, and atypia of glia and neurons (68, 69).

### 3.5 Chemotherapy-induced cognitive impairment

BM usually occurs late during metastatic BC. Therefore, patients with breast cancer brain metastases may receive up to several lines of chemotherapy before radiotherapy in the brain area. It is known that chemotherapy also impairs cognitive functions, and breast cancer patients are more sensitive to the symptoms of so-called “chemobrain”. One of the reasons often mentioned for this is the lack of other symptoms of the disease in patients with breast cancer and greater focus on cognitive disorders. Processing speed and attention span were shown to decrease in patients with stage I-IIIa breast cancer during chemotherapy and did not return to normal 2 months after systemic treatment (70). Breast cancer patients treated with intracranial radiotherapy for brain metastases are at risk for the cumulative adverse effects of several treatments on cognition.

## 4 Neurotoxicity-sparing strategies

The current understanding of BM biology and molecular underpinnings will facilitate the implementation of neuroprotective treatment protocols. The widespread use of brain RT has prompted the development of brain-sparing approaches. Available data were obtained from the studies performed in patients with various primary tumors. The most appealing treatment strategies include metastasis-directed RT (SRS/FSRT as previously described), hippocampus-avoiding (HA) radiation, and the use of memantine (12, 71–73). Effective systemic treatment of BCBM, which would allow clinicians to delay the use of intracranial radiotherapy, is still being researched, as well as completely novel approaches changing the paradigms of BCBM therapy.

### 4.1 Hippocampal-avoiding radiotherapy

**Figure 1:**
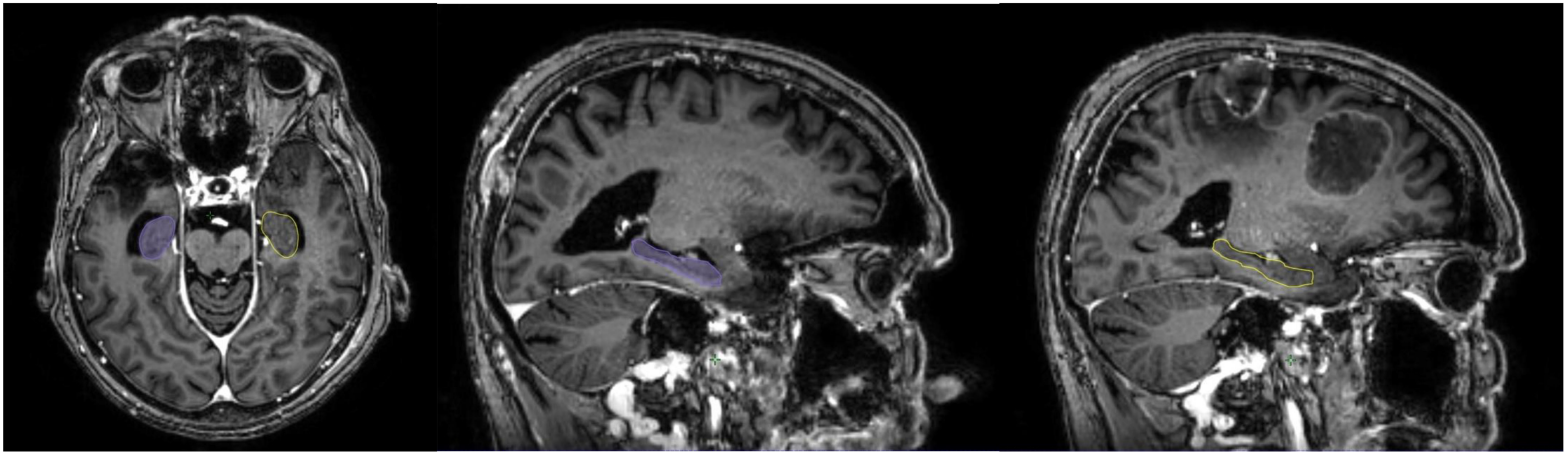
MR image with hippocampus contouring.

**Figure 2:**
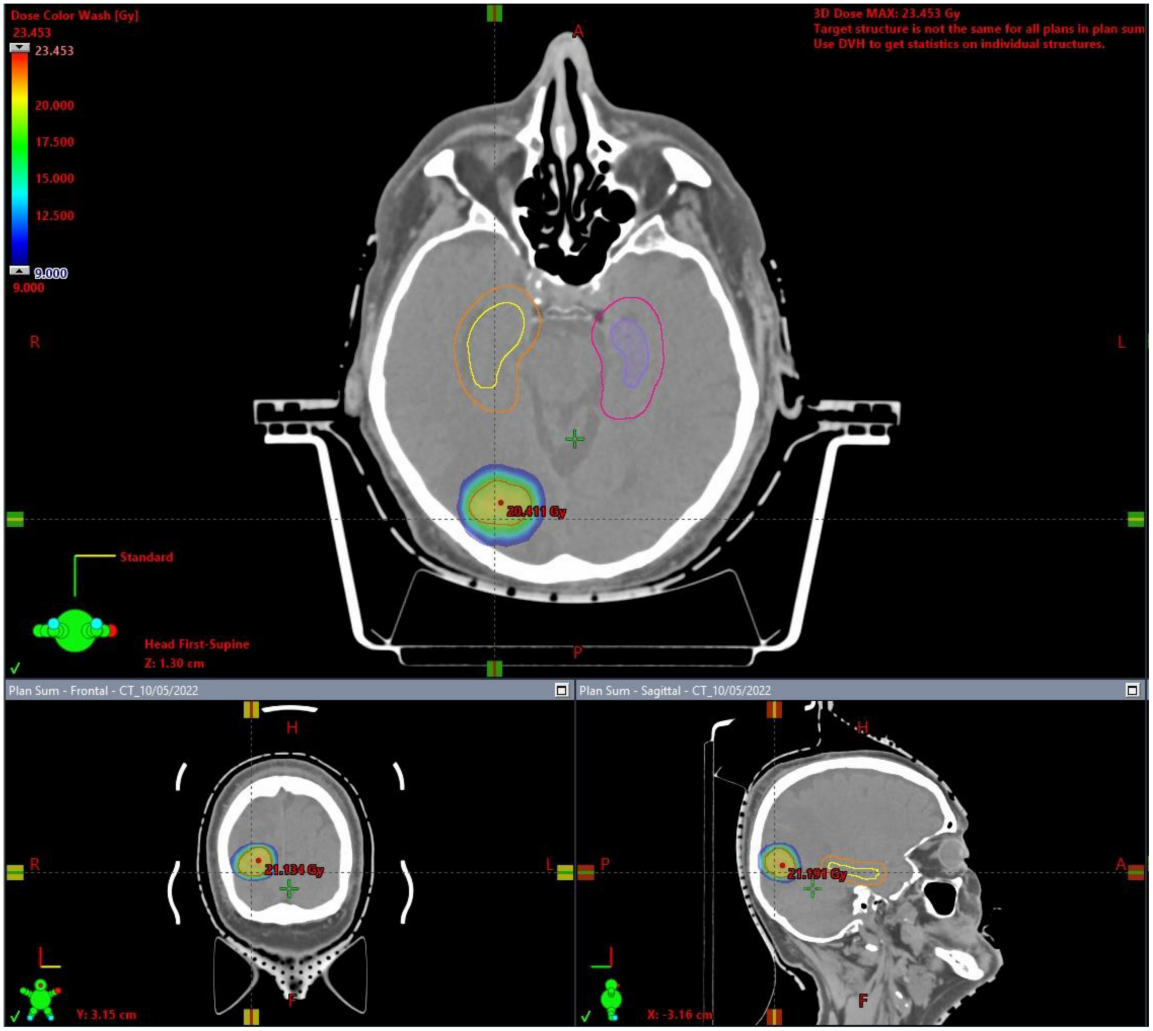
Hippocampal avoiding fractionated stereotactic radiotherapy (HA-FSRT) plan.

The hippocampus is essential in memory function. Neuronal progenitor cells of the hippocampal dentate gyrus, which are responsible for neurogenesis, are extremely vulnerable and radiosensitive. Neurogenesis is essential for the recovery of radiation-related loss memory and CF deterioration after WBRT (74–76). Interestingly, BM distribution correlates with the primary tumor diagnosis: BMs due to pulmonary and gastrointestinal cancers are usually located in the infratentorial area, BMs due to skin cancer and sarcoma are usually located in the supratentorial space, whereas BCBMs are located in structures supplied by the posterior circulation areas, such as the cerebellum (77). Several studies have assessed patterns of failure after SRS, showing 0-4.1% recurrences in the hippocampus (**Table 2**), justifying the HA approach in RT for BM (74, 78–84). The avoidance of the hippocampus during WBRT is considered to be safe, with approximately 7.6% and 12.1% perihippocampal disease progression after HA-WBRT (85–88). High-quality MRI is required to prepare the HA-SRS plan (**Figure 1**), limiting the doses within the hippocampus to D_mean_ <5 Gy for SRS and <7 Gy for FSRT (50) (**Figure 2**). The RTOG trial, the first phase II randomized study to prospectively assess an HA approach, showed a significantly lower incidence of verbal memory decline with an HA approach than without an HA approach (7% vs. 30%; p<0.001) (75), but BCBM patients accounted for only 15% of the study group, and cognitive function was assessed only for 6 months after the completion of radiotherapy. With an HA approach, it was possible to restrict the dose to the hippocampus to 9 Gy in 98% of the volume and to 17 Gy in 2% of the volume with no deterioration of treatment efficacy (89). Recently, the HA concept was evaluated in patients with small cell lung cancer (SCLC) receiving 25 Gy in 10 fractions as standard prophylactic cranial irradiation (PCI). In that phase III trial, 150 patients with SCLC (71.3% with limited disease) were randomized to standard PCI or HA-PCI (90). Data including delayed free recall (DFR) on the Free and Cued Selective Reminding Test (FCSRT), quality of life, the incidence and location of BM, and OS were collected at baseline and at 3, 6, 12, and 24 months after RT. The decline in DFR from baseline to 3 months was lower in the HA-PCI arm (5.8%) than in the PCI arm (23.5%; odds ratio, 5; 95% CI, 1.57-15.86; p=0.003). The analysis of all FCSRT scores showed a decline in the total recall (TR) (TR: 8.7% vs. 20.6%) at 3 months; DFR (11.1% vs. 33.3%), TR (20.3% vs. 38.9%), and total free recall (14.8% vs. 31.5%) at 6 months, and TR (14.2% vs. 47.6%) at 24 months. The incidence of BMs, OS, and quality of life were not significantly different between the groups. The researchers concluded that sparing the hippocampus is possible and facilitates CF preservation without deteriorating the outcomes of patients with SCLC. It is known that the clinical and molecular characteristics of BCBM patients are significantly different from those of SCLC patients receiving PCI. Clinical studies have not confirmed the benefit of PCI in patients with BC and have reported no evidence of cognitive dysfunction in PCI patients (91). Considering these reports and the fact that patients with BCBM may have increased symptoms of “chemobrain” after previous treatment, cognitive impairment associated with current brain metastases, and possible systemic treatment options active in brain metastases, translating the results of SCLC patients’ treatment to patients with BCBM seems difficult or impossible.

**Table 2:** Incidence of perihippocampal metastases.

Reference	Number of patients	Primary tumor	Perihippocampal (PH) metastases
**Gondi et al. (** 75)	**100**	**Lung cancer (56 pts)** **Breast cancer (15 pts)** **Other cancers (29 pts)**	**3 pts of 67 pts with intracranial progression had metastases in hippocampal-avoiding (HA) area after HA-WBRT (4,5%)**
**Hong et al. (** 78) **ANZMTG 01.07 WBRTMel**	**77** **(115 mets)**	**Melanoma**	**0 mets in hippocampus** **4 pts with PH metastases (5,2%)** **Median distance from the nearest hippocampal area was 37,2 mm** **Total volume of metastases was a significant predictor for the risk of metastases within the HA area**
**Harth et al. (** 79)	**100**	**Non-small cell lung cancer (NSCLS)** **Small cell lung cancer (SCLC)**	**3% of pts had metastases inside the hippocampus** **SCLC patients with high rate of hippocampal metastases (18,2% of all SCLC pts) vs. 2,8% in NSCLC**
**Ghia et al. (** 80)	**100** **(272 mets)**	**-**	**9 metastases within 5 mm from hippocampus (3,3% of mets; 8 pts)** **86,4% mets >15 mm from hippocampus**
**Wan et al. (** 81)	**488** **(2270 mets)**	**Non-small cell lung cancer (NSCLC) 58%** **Small cell lung cancer (SCLC) 9%** **Breast cancer 18%** **Other cancers (prostate, esophageal, renal, gynecological, colon, transitional and musculoskeletal cancers, melanoma) 15%**	**23 (4,7%) patients/25 (1,1%) metastases in:** **- Hippocampus (7pts/1,4%; 7 mets/0,3%)** **- Subventricular zone [SVZ] (18pts/3,7%; 18 mets/0,8%)** **Only 1/7 metastasis located in the hippocampus occurred in oligometastatic patients; 6/7 metastases located in the hippocampus and all 18 metastases located in the SVZ occurred in nonoligometastatic patients**
**Sun et al. (** 82)	**314** **(1678 mets)**	**Breast cancer**	**4,1% of pts had metastases in PH area (1,2% of all mets)** **Only the number of BMs was significantly correlated with PH disease in the multivariate analysis** **The risks of PH metastasis recurrence were 4.6% for WBRT and 6.8% for subtherapeutic irradiation in the PH region (the increase was approximately 2%)**

Nevertheless, due to the lack of research results on the use of HA-WBRT in the BCBM group, considering the results of the cited studies, the increasing availability of MRI and the relatively small group of patients with BCBM not eligible for SRS/FSRT, we often use the HA-WBRT technique in everyday clinical practice as we describe in detail later in the work.

### 4.2 Memantine

In a randomized, double-blind, placebo-controlled RTOG 0614 trial, adult patients with a BM outside the 5 mm margin around the hippocampus and a KPS score >70 were randomly allocated to WBRT or WBRT with the addition of memantine (92). Memantine has been used as an uncompetitive antagonist of the *N*-methyl-D-aspartate (NMDA) receptor, which is crucial for synaptic plasticity, a cellular mechanism of learning and memory. Memantine administered concomitantly with WBRT was shown to be beneficial. Cognitive decline was delayed (HR=0.78, 95% CI, 0.62-0.99, p=0.01), while memory decline was less at 24 weeks, but it was not statistically significant (p=0.059). Superior results were also seen for memantine in executive function at 8 weeks (p=0.008), processing speed at 24 weeks (p=0.0137) and delayed recognition at 24 weeks (p=0.0149). Overall, the probability of cognitive function failure at 24 weeks in the memantine arm was 53.8%, while 64.9% in the placebo arm. In NRG COO1, HA-WBRT combined with memantine significantly improved CF (HR=0.74, 95% CI, 0.58–0.95; p=0.020) over WBRT plus memantine, showing promising results for combined treatment (71). However, patients with BCBM constituted less than 15% in the RTOG 0614 study and 18.5% in the NRG CC001 trial, and the observation period was only 6-8 months after radiotherapy, which is a limitation of these trials. Apart from the heterogeneous histology of BM, other limitations of these studies include the lack of information on the systemic treatment used and the number and initial volume of BM, as well as on comorbidities or cigarette smoking, which could have influenced cognitive functions. There are several reports showing that despite the favorable results of clinical trials and the potentially simple implementation of memantine treatment into clinical practice, few radiotherapists recommend its use (93–95). A recent update of the NRG CC001 trial was published with a median follow-up of 12.1 months, showing sustained preservation of cognitive function in the HA-WBRT + memantine arm (adjusted hazard ratio, 0.74, P = .016) (96). Patients who received HA-WBRT + memantine experienced less symptom burden at 6 (P <.001 using imputed data) and 12 months (P = .026 using complete-case data; P <.001 using imputed data), less symptom interference at 6 (P = .003 using complete-case data; P = .0016 using imputed data) and 12 months (P = .0027 using complete-case data; P = .0014 using imputed data), and fewer cognitive symptoms over time (P = .043 using imputed data). There were no differences in overall survival, intracranial progression-free survival, or toxicity between treatment arms.

Thus, confirmation of the beneficial neuroprotective effect of memantine in BCBM is needed, but considering the availability of the therapy, its relatively low cost and low toxicity, treatment with memantine may be offered to patients with BCBM who cannot avoid WBRT, and especially to those who are determined to undergo neurotoxicity-sparing treatment.

### 4.3 FLASH radiotherapy (FLASH-RT)

FLASH-RT is a new option in external-beam therapy. It appeared in the literature in 1960-1970, but it was rediscovered in 2014 (97). In FLASH-RT, single short radiotherapy pulses are delivered at ultrahigh dose rates >40 Gy/s as opposed to conventionally used rates of 0,07-0,1 Gy/s. This technique has gained interest due to unexpected normal tissue tolerance with efficacy similar to conventional radiotherapy. Montay-Gruel et al. (98) have demonstrated the sparing of memory in mice after whole brain irradiation of a single dose of 10 Gy when dose rates above 100 Gy/s were achieved. This effect (called the FLASH effect) was significantly lower in the range of 30-100 Gy/s and disappeared completely with dose rates <30 Gy/s. The researchers revealed the preservation of memory and neurogenesis in the hippocampus 2 months after FLASH radiotherapy, but these results were also found after 6 months of observation. Additional experiments showed lower levels of reactive oxygen species (ROS), a lack of neuroinflammation and dendritic complexity and a synaptic landscape similar to the nonirradiated brain tissue (99).

In the context of breast cancer treatment, FLASH radiotherapy is also being studied, but mainly in the context of adjuvant radiotherapy after breast cancer surgery, and most of these studies involve animal models or feasibility studies in humans (100–102).

Although FLASH radiotherapy has been shown to improve the therapeutic index, especially in brain irradiation, most of the knowledge was obtained from animal studies, and a better understanding of the radiobiology of this new therapy is still needed. Clinical implementation will also require solutions to technical challenges, but the investigators think that progress from achievements made during the last decade is just a matter of time.

### 4.4 Systemic treatment of BCBM

Anaplastic lymphoma kinase (ALK)-mutated or epidermal growth factor receptor (EGFR)-mutated lung adenocarcinoma patients with brain metastases are primarily treated with systemic molecular therapies instead of upfront brain irradiation, especially when there are few and small lesions in the CNS. This therapeutic change became possible due to high BBB penetration by new anti-ALK and anti-EGFR drugs. It is still uncertain whether primary systemic treatment in the case of BCBM will delay the application of intracranial radiotherapy and thus delay its toxicity.

As mentioned above, BCBM patients are usually excluded from clinical trials or are a small group in the treated population, making it impossible to draw conclusions about recommended therapy. Currently, according to the growing population of surviving advanced breast cancer patients, there are multiple ongoing clinical trials in disseminated BC investigating new agents alone or in combination with already well-established treatments (such as trastuzumab, lapatinib, and capecitabine). Of the most studied drugs are inhibitors of the PI3K/AKT/mTOR (phosphatidylinositol-3 kinase/AKT/mammalian target of rapamycin) signaling pathway, HER2 (human epidermal growth factor receptor 2) and pan-HER inhibitors, immunotherapy, CDK4/6 (cyclin-dependent kinases 4/6) inhibitors and PARP (polyadenosine diphosphate-ribose polymerase) inhibitors.

The PI3K/AKT/mTOR pathway is activated in 30-40% of BCs and even in 43-75% of BCBMs, making this molecular disorder a potential therapeutic target (103). The PI3K/AKT/mTOR inhibiting regiment of buparlisib plus capecitabine is being tested in an ongoing clinical trial (NCT02000882).

Trastuzumab, an anti-HER2 antibody, is too large to penetrate the BBB but is very effective extracranially and prolongs the survival of BC patients; however, it increases the risk of metastasis to the CNS as the first relapse location (104). The subsequently developed anti-HER2 antibody pertuzumab showed disappointing results against brain metastases (105). The regimen of lapatinib (dual tyrosine-kinase inhibitor [TKI] of HER1 and HER2) combined with capecitabine is effective in HER2+ BCBM (106), with a median time to WBRT of 8.5 months in untreated low volume BM (107). Trastuzumab conjugates (trastuzumab-emtansyna [TDM-1] and trastuzumab-deruxtecan [T-Dxd]) showed promising activity in the CNS, especially T-Dxd (42, 108–110). Ultimately, in search of small molecules that easily penetrate the blood−brain barrier, novel TKIs inhibiting the human epidermal growth factor receptor family have been developed (tucatinib, neratinib, pyrotinib), showing promising effectiveness in HER2+ BCBM (111–113). Nevertheless, there are many ongoing trials to establish their role in clinical practice, also in the context of brain irradiation, as it is uncertain if they should be used before, after or even with CNS radiotherapy as radiosensitizers (114).

The role of immunotherapy in the setting of BCBM is less certain, as it shows positive results only in metastatic TNBC; however, no benefit has been found in the subgroup of patients with BCBM (115). The knowledge in this subject is limited, but the results of a few trials are on the way, including those of studies combining immunotherapy with brain radiotherapy (NCT03449238 and NCT03483012).

More optimistic results have been seen in HR+ HER2- BCBM patients treated with CDK4/6 inhibitors, particularly abemaciclib, which showed good penetration through the BBB (116). Prospective trials are underway (NCT02308020, NCT02896335, and NCT04334330).

PARP inhibitors (olaparib and talazoparib) already approved by the FDA for the treatment of germline BRCA-mutated BC have been investigated in BCBM. To date, talazoparib (117) and veliparib (118) have shown activity in the brain, interestingly in the case of veliparib in combination with WBRT, but further studies are expected to elucidate their role in BCBM and the optimal sequence of treatment modalities.

### 4.5 Secondary prevention of BCBM

SRS/FSRT is an effective method of local treatment for BCBM, with fewer local failures than surgical treatment and lower neurotoxicity than WBRT due to the reduction in the irradiated brain volume, including the uninvolved hippocampus. At the same time, limiting treatment to existing metastases carries the risk of metastasis development in the rest of the brain. WBRT reduces this risk at the expense of neurotoxicity without prolonging survival (**Table 1**). A new idea is to prevent the development of secondary BCBM (after initial local therapy) with systemic treatment. In preclinical studies in mice, at low metronomic doses, temozolomide (TMZ), a BBB-crossing alkylating agent routinely used in the treatment of glioblastoma, has been shown to reduce the risk of developing brain metastases, despite its lack of efficacy in treating existing metastases. In a phase 1 study, metronomic use of TMZ in combination with T-DM1 showed low toxicity and potential activity in the secondary prevention of BCBM in HER2(+) BC patients (119). A phase II study evaluating this treatment is ongoing (NCT03190967).

### 4.6 Other options

There are many other methods of treating BCBM under investigation that avoid radiotherapy and its toxicities. Many of them are incorporating new approaches to omit the problem with the BBB. One of them is intrathecal administration of drugs, such as trastuzumab (120). The second emerging technology is nanotherapy (121), which uses nanoparticles carrying anticancer agents to deliver drugs, but further research on its role in BCBM is needed. The other option includes brain radiotherapy to improve the penetrability of the BBB for systemic treatment, but its role in this capacity has not been well proven.

Another interesting possibility is liquid biopsy of cerebrospinal fluid to identify the exact molecular alterations of BCBM cells (122), as it is well known that metastatic breast cancer cells can change their molecular features during invasion and metastasis formation, for instance, in the case of HER2 amplification. Establishing the molecular status of brain metastases can be used to tailor treatment more precisely and effectively.

## 5 Practical implications

The group of patients with BCBM is not homogeneous. The choice of BM treatment method is determined by the patient’s age, performance status, number, size and location of brain metastases and control of extracranial metastases.

Traditionally patients with a single brain metastasis, especially over 3 cm, with neurosurgical symptoms, severe edema and mass effect and/or located in the posterior cranial fossa with hydrocephalus, were eligible for neurosurgical treatment, if the extracranial metastases were stable and systemic treatment options were available.

In everyday practice, we increasingly qualify patients with BCBM for primary BM radiotherapy due to the increased risk of dissemination to the meninges after primary neurosurgical treatment, which in patients with TNBC may be as high as 24%. The qualification criteria for SRS/FSRT include the potential possibility of further systemic treatment, the slow dynamics of the disease, previous intracranial radiotherapy, and the patient’s general condition. If at least 2 out of 4 factors favor SRS/FSRT, we qualify patients for this method, even in the presence of >4 metastatic foci if they are countable. Traditionally, we qualified patients with a single BCBM <= 2 cm for SRS; currently, the final choice of irradiation technique is determined by the parameters of the radiotherapy plan. To diagnose intracranial spread, we use 3-Tesla magnetic resonance imaging using a SPACE sequence, thanks to which we detect more small lesions. If, when preparing the radiotherapy plan, the measured volume of metastases exceeds 25 cm3, it will not be possible to safely and effectively perform SRS and we qualify the patient for FSRT. In case of BCBM volume <25cm3, we prepare SRS. If the irradiation plan meets the radiosurgical criteria, i.e. with a dose of approximately 20Gy in 1 fraction for metastatic lesions, the volume of the healthy brain receiving a dose of 12Gy is less than 5 cm3 (V12<5 cm3) and the plan conformity index is <1.4 (CI<1, 4), we qualify the patient for SRS. If the radiotherapy plan does not meet the above parameters, we qualify the patient for FSRT, usually 27Gy in 3 fractions or 30/35Gy in 5 fractions, depending on meeting the remaining constraints for intracranial radiotherapy, including the average dose to the hippocampus below 7 Gy (Dmean<7Gy). Patients with poor general condition, short expected survival, lack of cooperation with medical staff and uncontrolled epilepsy are not eligible for SRS/FSRT.

For patients with BCBM who are not eligible for SRS/FSRT, e.g. due to uncountable brain metastases, or in the case of BCBM from TNBC undergoing neurosurgery, remaining in good general condition, cooperating with medical staff, in whom we expect > 3-month survival, we use WBRT. In each case in which we have available brain MRI, we use HA-WBRT, using 30Gy in 10 fractions (12 days of treatment), with the maximum dose (the highest dose in 0.03 cm3 of both hippocampi) limited to 16Gy [Dmax <=16Gy], and doses in the entire hippocampal volume up to 9 Gy [D100% <=9Gy]. Approximately 3 days before starting HA-WBRT, we start memantine. In the first week at a dose of 1 x 5mg orally in the morning, followed by the addition of a 5-mg dose in the evening during week 2. In week 3, we increase the morning dose to 10 mg. In the fourth week we reach a dose of 2 x 10 mg per day and continue this treatment for up to 24 weeks in total. In case of creatinine clearance below 30 mL/min the maximum dose is 5 mg orally twice daily and the treatment should not be administered if the creatinine clearance is less than 5 mL/min.

According to the previously cited clinical trial data, we know that both the metastatic changes themselves and oncological treatment, both with radiotherapy and systemic therapy, have a significant impact on the cognitive functions of a patient with BCBM. Basic screening tests such as MMSE have limited sensitivity in detecting these disorders, and oncologists and radiation oncologists in their daily practice do not have enough time or knowledge to perform more advanced tests. Due to the popularization of Breast Units, which comprehensively deal with the treatment of patients with breast cancer and bring together doctors of various specialties, senology nurses, dieticians and psychologists in one place, it seems reasonable that patients with BCBM should also be provided with care by neuropsychologists. This would allow us to obtain real-world data about the frequency, depth and persistence of cognitive disorders in BCBM patients and their dependence on oncological treatment.

## 6 Future directions

HA-WBRT +/- memantine is not routine practice in radiotherapy facilities, despite phase III studies showing neurocognitive benefits and safety of such therapy. One of the reasons is the heterogeneity of the patient population included in these studies. Another criticism was short follow-up, but the recently published update of the NRG CC001 study demonstrated that the benefits persisted at one-year follow-up. Another reason is the mediocre benefit of the intervention - in the group of patients treated with HA-WBRT, 60% still have cognitive disorders after 6 months. There are also technical and economic aspects. WBRT can be planned and implemented easily in any radiotherapy facility, even within 5 days. HA-WBRT requires the use of MRI to contour the hippocampi and the use of intensity-modulated beam technique (IMRT) by physicists, and the usual regimen is 30 Gy in 10 fractions, which extends the procedure time to 2-3 weeks and significantly increases workload on par with SRS/FSRT planning. In some countries, despite the same amount of work, the HA-WBRT procedure may be less cost-effective than SRS/FSRT. For this reason, the results of a study comparing SRS with HA-WBRT + memantine in less than 5 BM may provide a solution for everyday practice (NCT03550391). We expect an increase in the use of stereotactic techniques in the treatment of BCBM, which will replace surgical treatment associated with a higher risk of meningeal metastases and allow for dose reduction not only in the hippocampus, but also in other parts of the healthy brain related to cognitive functions, such as the corpus callosum. Technological development now allows for safe and effective irradiation of patients with > 10 BM with FSRT.

Secondly, the radiotherapy community also expects the results of studies assessing the effectiveness of systemic treatment in BCBM, especially in HER2+ breast cancer, because small molecules with good penetration of the BBB may change the paradigm and replace intracranial radiotherapy or significantly delay its use. Old radiosensitizers have failed in the treatment of BM, but we expect that intracranial radiotherapy combined with modern hormone therapy, immunotherapy or PARP inhibitors will change the standards of management of BCBM.

## 7 Conclusions

Despite the significant incidence rate, patients with brain metastases from breast cancer (BCBMs) remain underrepresented in randomized clinical trials (RCTs) that investigate diverse strategies for minimizing cognitive deficits after intracranial radiotherapy. The bulk of available data originates from studies involving lung cancer patients, particularly those with non-small cell lung cancer (NSCLC). Given the disparities in clinical and molecular attributes along with variations in prognosis between NSCLC and distinct subtypes of breast cancer it becomes imperative to validate these findings within a cohort of BCBM (123, 124). This validation should consider the long-term repercussions of approaches aimed at mitigating radiation-induced neurotoxicity, in contrast to the prevailing trend of evaluating outcomes only at 6-month juncture, as observed in prior trials.

The augmented survival rate among breast cancer patients with well-regulated systemic control presents an opportunity to employ an efficacious and safe method for managing intracranial disease. Emerging systemic therapies, particularly those tailored for HER2-positive breast cancer, exhibit enhanced potency in managing brain metastases. This development may potentially defer the need for immediate radiotherapy in BCBMs (111, 125). However, for those with triple-negative breast cancer (TNBC) BCBMs, the optimal synergy between systemic treatment and intracranial radiotherapy - both in terms of efficacy and safety – remains uncertain. The anticipation of utilizing immunotherapies and PARP inhibitors for this indication is on the horizon.

In tandem with these advancements, novel treatment modalities like FLASH radiotherapy, nanoparticles facilitating drug delivery to the brain, and TMZ as the secondary prevention for HER2+ BCBM following stereotactic radiosurgery (SRS) and/or surgery, are subjects of intensive research.

These innovations have the potential to reshape the fundamental approaches to treat BCBMs. It is crucial to underscore that expecting comprehensive human data encompassing anatomopathological and molecular changes linked to radiotherapy is an impracticable endeavor. To synthesize drawing upon existing insights derived from animal studies concerning radiation-induced brain damage, along with strategies aimed at alleviating neurotoxicity in human cases, the suggestion is to prioritize stereotactic radiosurgery (SRS) or fractionated stereotactic radiotherapy (FSRT) over whole-brain radiotherapy (WBRT) when addressing patients with oligometastatic brain metastases from breast cancer (BCBMs). Even in cases of polymetastatic BCBMs, that can be enumerated, FSRT stands as the preferred approach. In scenarios when WBRT is unavoidable, such as when facing uncountable number of BCBMs or in cases of surgically resected triple-negative BCBMs, hippocampal-avoidant WBRT (HA-WBRT) in conjunction with memantine administration may be considered.

## Author contributions

All authors listed have made a substantial, direct, and intellectual contribution to the work, wrote the manuscript and approved it for publication.
